# Gut microbiota alterations in critically Ill patients with carbapenem-resistant Enterobacteriaceae colonization: A clinical analysis

**DOI:** 10.3389/fmicb.2023.1140402

**Published:** 2023-04-04

**Authors:** Moon Seong Baek, Seungil Kim, Won-Young Kim, Mi-Na Kweon, Jin Won Huh

**Affiliations:** ^1^Department of Internal Medicine, Chung-Ang University Hospital, Chung-Ang University College of Medicine, Seoul, Republic of Korea; ^2^Department of Convergence Medicine, Asan Medical Center, University of Ulsan College of Medicine, Seoul, Republic of Korea; ^3^Department of Pulmonary and Critical Care Medicine, Asan Medical Center, University of Ulsan College of Medicine, Seoul, Republic of Korea

**Keywords:** carbapenem-resistant Enterobacteriaceae (CRE), microbiome, intestinal dysbiosis, short-chain fatty acid, metabolic networks and pathways

## Abstract

**Background:**

Carbapenem-resistant Enterobacteriaceae (CRE) are an emerging concern for global health and are associated with high morbidity and mortality in critically ill patients. Risk factors for CRE acquisition include broad-spectrum antibiotic use and microbiota dysbiosis in critically ill patients. Therefore, we evaluated the alteration of the intestinal microbiota associated with CRE colonization in critically ill patients.

**Methods:**

Fecal samples of 41 patients who were diagnosed with septic shock or respiratory failure were collected after their admission to the intensive care unit (ICU). The gut microbiota profile determined using 16S rRNA gene sequencing and quantitative measurement of fecal short-chain fatty acids were evaluated in CRE-positive (*n* = 9) and CRE negative (*n* = 32) patients. The analysis of bacterial metabolic abundance to identify an association between CRE acquisition and metabolic pathway was performed.

**Results:**

CRE carriers showed a significantly increased proportion of the phyla Proteobacteria and decreased numbers of the phyla Bacteroidetes as compared to the CRE non-carriers. Linear discriminant analysis (LDA) with linear discriminant effect size showed that the genera *Erwinia*, *Citrobacter*, *Klebsiella, Cronobacter*, *Kluyvera*, *Dysgomonas*, *Pantoea*, and *Alistipes* had an upper 2 LDA score in CRE carriers. The alpha-diversity indices were significantly decreased in CRE carriers, and beta-diversity analysis demonstrated that the two groups were clustered significantly apart. Among short-chain fatty acids, the levels of isobutyric acid and valeric acid were significantly decreased in CRE carriers. Furthermore, the PICRUSt-predicted metabolic pathways revealed significant differences in five features, including ATP-binding cassette transporters, phosphotransferase systems, sphingolipid metabolism, other glycan degradation, and microbial metabolism, in diverse environments between the two groups.

**Conclusion:**

Critically ill patients with CRE have a distinctive gut microbiota composition and community structure, altered short-chain fatty acid production and changes in the metabolic pathways. Further studies are needed to determine whether amino acids supplementation improves microbiota dysbiosis in patients with CRE.

## 1. Introduction

Nosocomial multidrug-resistant organism (MDRO)-induced infections constitute a major problem in intensive care units (ICU) worldwide (Vincent et al., 2009). Methicillin-resistant *Staphylococcus aureus* (MRSA), vancomycin-resistant *Enterococcus* (VRE), multidrug resistant *Acinetobacter baumannii* (MRAB), and extended-spectrum β-lactamases producing Enterobacteriaceae (ESBL-E) are the organisms that commonly cause nosocomial infections (Zaragoza et al., 2020). However, carbapenem-resistant Enterobacteriaceae (CRE) has recently emerged as a global challenge because of the high CRE-associated morbidity and mortality rates (Falagas et al., 2014). CRE infections are difficult to treat because of extensive resistance patterns and the limited effective antimicrobial therapeutic options (Magiorakos et al., 2017). Several factors, including prior antimicrobial exposure, longer ICU stay, or residence in a long-term care facility, contribute to the occurrence of CRE (Yi and Kim, 2021). The presence of CRE colonization at ICU admission is associated with higher CRE infection-associated 30- and 90-day mortality risks (McConville et al., 2017). Furthermore, the prevention of CRE transmission incurs a substantial economic burden. In a surgical ICU, the financial costs accrued from CRE outbreaks in 3 years was approximately €1,300,000 (Atchade et al., 2022). Therefore, decreasing the risk for CRE acquisition and ensuring active surveillance for the detection of CRE carriers is essential in the ICU (Yi and Kim, 2021).

The identification of gut microbiota such as next-generation sequencing or 16S rRNA profiling has substantially improved the understanding and insight into the composition and function of the gut microbiota (Langille et al., 2013; Zeng et al., 2017; Szychowiak et al., 2022). In the gut microbiome, the abundant phyla are Firmicutes (60–75%), Bacteroidetes (30–40%), Actinobacteria, and Proteobacteria (Arumugam et al., 2011). The normal microbiota confers protection against the enteric pathogens, including MDROs, and decrease the colonization pressure (Buffie and Pamer, 2013). However, the use of antibiotics or ICU-specific therapies, such as selective digestive decontamination, can result in a state of disequilibrium of the microbiota (Szychowiak et al., 2022). This dysbiosis of microbiota in critically ill patients is associated with a poor prognosis and/or an increased risk for infection. Furthermore, short-chain fatty acids, which are produced by the bacterial fermentation of undigested carbohydrates (Cummings et al., 1987), play a crucial role as the major energy source, ensure the maintenance of the barrier function in the colonic epithelium, and help regulate the immune system (Maslowski and Mackay, 2011).

We hypothesized that the gut microbiota, the related short-chain fatty acids, or multiple metabolic pathways differed between CRE carriers and CRE non-carriers. In this study, we aimed to determine the differences in the intestinal microbiota according to the identification of the CRE status of critically ill patients.

## 2. Materials and methods

### 2.1. Study design and eligible patients

This retrospective study was conducted at Asan Medical Center, which is a tertiary hospital with 2,800 beds in the Republic of Korea. Between 1 October 2016, and 31 August 2021, adult critically ill patients (age >18 years) who were diagnosed with septic shock or respiratory failure were eligible for inclusion. Patients who were admitted to the ICU with the abovementioned diagnoses were requested to provide a stool sample after obtaining written informed consent for participation in the study. This study was approved by the Institutional Review Board (IRB) of Asan Medical Center (IRB 2011-0001), and was conducted in accordance with the Declaration of Helsinki as well as the local regulations governing clinical studies.

### 2.2. Data collection and definitions

The following data were retrospectively obtained from the electronic medical records of the participants: demographic data, including age, sex, body mass index, smoking status (current smoker, ex-smoker, and never smoker), comorbidities (hypertension, diabetes, chronic lung disease, chronic kidney disease, chronic liver disease, cardiovascular disease, neurologic disorder, solid tumor, and hematologic malignancy), and gastrointestinal symptoms. Additionally, clinical data at hospitalization, such as hospitalization history within 1 year, and medication history (chemotherapy or immunosuppressant use, antibiotic therapy, and digestive therapy) within the preceding 3 months were obtained. Furthermore, data pertaining to the ICU admission, including the interval between hospitalization to ICU admission, route of ICU admission (emergency department, ward, and other hospital), cause of ICU admission (septic shock or respiratory failure), severity scores [Acute Physiology and Chronic Health Evaluation (APACHE II) and Sequential Organ Dysfunction Assessment (SOFA) scores] at ICU admission, mechanical ventilation, renal replacement therapy, length of ICU stay, duration of mechanical ventilation, length of hospital stay, and the in-hospital mortality rate, were collected.

At the time of fecal sampling, the following variables were collected: interval from hospitalization to sampling, interval from ICU admission to sampling, diet (nil *per os* and enteral nutrition), and medications (e.g., opioid, sedatives, norepinephrine, vasopressin, probiotics, H_2_ blocker, proton-pump inhibitor, antibiotics, and antifungal agents) within the preceding 7 days. Moreover, infection-related variables, including bacteremia, *Clostridium difficile* infection (CDI), aspergillosis, and the MDROs (ESBL-E, MRAB, VRE, MRSA, and CRE) that were identified within 1 month, were collected.

Carbapenem-resistant Enterobacteriaceae are defined as Enterobacteriaceae that are resistant to any carbapenem antibiotics (i.e., ertapenem, meropenem, doripenem, or imipenem), and include both non-carbapenemase-producing Enterobacteriaceae (non-CP-CRE) and carbapenemase-producing Enterobacteriaceae (CP-CRE) (Magiorakos et al., 2017). We defined CRE carriers as participants in whom CRE was detected in any type of specimen, including blood, sputum, urine, stool, or rectal swab, regardless of the presence of CRE-related symptoms (Magiorakos et al., 2017). Based on the Sepsis-3 definition, septic shock was defined based on the requirement of vasopressors to maintain a mean arterial pressure of 65 mmHg or higher and lactic acid 2 mmol/L or higher in patients with sepsis (Singer et al., 2016). Respiratory failure included gas-exchange dysfunction due to a respiratory system failure, which was characterized as hypoxemic (PaO_2_ <60 mmHg) and/or hypercapnic (PaCO_2_ >45 mmHg) respiratory failure (Roussos and Koutsoukou, 2003). The diagnosis of CDI was established in patients with diarrhea based on a positive result of the stool test for toxigenic *C. difficile* or its toxins, or from colonoscopic/histopathologic findings of pseudomembranous colitis (Bagdasarian et al., 2015).

### 2.3. Microbiome analysis

Fecal samples of participants (*n* = 48) were collected, and we excluded fecal samples (*n* = 7) that did not meet the analytical quality. Stool samples were transported to the laboratory and stored at −80°C. Total DNA was extracted from the feces by using QIAamp Fast DNA stool mini kits (Qiagen) in accordance with the manufacturer’s instructions. Primers 341F and 805R that target bacterial 16S rRNA gene were used for bacterial PCR amplification, and the amplified products were purified and sequenced by Chunlab (Seoul, Republic of Korea) with an Illumina Miseq Sequencing System (Illumina). The processing of raw reads started with a quality check and the filtering of low-quality reads (<Q25) using Trimmomatic ver. 0.32 (Bolger et al., 2014). After a quality control pass, the paired-end sequence data were merged using the VSEARCH version 2.13.4 and default parameters. Non-specific amplicons that did not encode 16S rRNA were detected by the “nhmmer” program in the HMMER package ver. 3.2.1 (Wheeler and Eddy, 2013). We used the EzBioCloud 16S rRNA database for taxonomic assignment using precise pairwise alignment (Yoon et al., 2017). After chimeric filtering, reads that were not identified to the species level (with <97% similarity) in the EzBioCloud database were compiled. Operational taxonomic units with single reads (singletons) were omitted from further analysis. The alpha and beta diversities were estimated for ascertaining the difference in samples. A taxonomic cladogram was generated using linear discriminant effect size (LEfSe) with a threshold of 2 on the logarithmic linear discriminant analysis (LDA) score (Segata et al., 2011).

### 2.4. Quantitative measurement of short-chain fatty acids

All reagents and solvents for metabolite analysis were purchased from Sigma. Freeze-dried feces (10 mg) were homogenized vigorously with 400 μl internal standard solution [1 mM propionic acid (C_3_)-d_6_, 100 μM butyric acid (C_4_)-d_7_, 100 μM of valeric acid (C_5_)-d_4_, 100 μM of Hexanoic acid (C_6_)-d_5_ in water] and centrifuged with 13,200 rpm for 10 min (Song et al., 2019). After centrifugation, the supernatant was filtered out. Then, 20 μl 20 mM AABD-SH, 20 μl 20 mM TPP, and 20 μl DPDS in dichloromethane were added to the filtrate. The solution was incubated for 10 min at RT with vortexing and thereafter vacuum-dried. The sample was reconstituted with 80 μl methanol prior to the LC-MS/MS analysis in an LC-MS/MS system equipped with a 1290 HPLC (Agilent Technologies, Denmark) Qtrap 5500 (ABSciex) and a reverse-phase column (Pursuit 5 C18 150 × 2.0 mm; Agilent Technologies). The extracted ion chromatogram (EIC), which corresponded to the specific transition for each metabolite, was used for quantitation. The area under the curve of each EIC was normalized to the internal standard. The peak area ratio of each metabolite was normalized to the internal standard using the feces weight in a sample, and the value obtained was then used for the comparison.

### 2.5. Prediction of metabolic pathway

To predict bacterial metabolic abundance and functional gene profiles defined by the Kyoto Encyclopedia of Genes and Genome (KEGG) pathway, Phylogenetic Investigation of Communities by Reconstruction of Unobserved States (PICRUSt) with the bacterial 16S rRNA gene sequences dataset was used (Langille et al., 2013). The statistically significant KEGG pathway of each group was identified by the LEfSe (LDA scores >2).

### 2.6. Statistical analysis

Statistical analyses were performed by using Prism software (GraphPad, La Jolla, CA, USA) and R ver. 4.1.2 (R Project for Statistical Computing) with the Fisher’s exact test and Mann–Whitney *U* test. Categorical variables are expressed as the number (percentage) whereas continuous variables are expressed as the median [interquartile range (IQR)]. Data are presented as the mean ± SD; *p* < 0.05 was considered statistically significant.

## 3. Results

### 3.1. Characteristics of the study population

Between 1 October 2016, and 31 August 2021, data from 41 critically ill patients were included in the analysis. This cohort included 9 CRE carriers (22%) and 32 non-carriers (78%). This cohort included 9 CRE carriers (22%) and 32 non-carriers (78%). Among the CRE carriers, the pathogens identified were seven cases with *Klebsiella pneumoniae*, one with *Escherichia coli*, and one with *Klebsiella oxytoca*. The cohort included five non-CP-CRE and four CP-CRE cases. All CP-CRE cases had the KPC enzyme. Baseline characteristics, including demographic data, are summarized in Table 1. The mean age of the participants in this cohort was 64 years (IQR 54–68.5) and 70.7% (*n* = 29) was male. The proportions of patients with a history of hospitalization within 1 year and of antibiotic therapy within 3 months were 58.5% (*n* = 24) and 34.1% (*n* = 14), respectively. The causes of ICU admission were septic shock in 46.3% (*n* = 19) and respiratory failure in 53.7% (*n* = 22) of the cohort. The median SOFA score at ICU admission was 12 (7–15), and mechanical ventilation was used in 90.2% (*n* = 37) of the cohort. The ICU and in-hospital mortalities were 22.0% (*n* = 9) and 36.6% (*n* = 15), respectively. Gastrointestinal symptoms were not significantly different between CRE positive and CRE negative group (44.4 vs. 62.5%, *p* = 0.450). The hospital stay before ICU admission was significantly longer in the CRE positive than in the CRE negative group.

**TABLE 1:** Baseline characteristics of the participants.

Variables	Total (*n* = 41)	CRE carriers (*n* = 9)	CRE non-carriers (*n* = 32)	*p*-value
Age (years)	64 (54–68.5)	64 (55–69)	64.5 (54–69)	0.801
Sex (male)	29 (70.7)	6 (66.7)	23 (71.9)	1.000
Body mass index (kg/m^2^)	22.7 (19.3–24.5)	23.0 (21.0–26.3)	22.7 (18.6–24.4)	0.244
Smoking status				1.000
Current smoker	7 (17.1)	1 (11.1)	6 (18.8)	
Ex-smoker	15 (36.6)	3 (33.3)	12 (37.5)	
Never smoker	19 (46.3)	5 (55.6)	14 (43.8)	
**Comorbidities**				
Hypertension	6 (14.6)	0 (0.0)	6 (18.8)	0.309
Diabetes	10 (24.4)	3 (33.3)	7 (21.9)	0.662
Chronic lung disease	12 (29.3)	3 (33.3)	9 (28.1)	1.000
Chronic kidney disease	5 (12.2)	2 (22.2)	3 (9.4)	0.299
Chronic liver disease	7 (17.1)	3 (33.3)	4 (12.5)	0.165
Cardiovascular disease	4 (9.8)	0 (0.0)	4 (12.5)	0.559
Neurologic disorder	2 (4.9)	0 (0.0)	2 (6.3)	1.000
Solid tumor	14 (34.1)	2 (22.2)	12 (37.5)	0.692
Hematologic malignancy	6 (14.6)	3 (33.3)	3 (9.4)	0.107
Gastrointestinal symptoms	24 (58.5)	4 (44.4)	20 (62.5)	0.450
Hospitalization history (<1 year)	24 (58.5)	8 (88.9)	16 (50.0)	0.056
Chemotherapy or immunosuppressant use (<3 months)	16 (30.9)	4 (44.4)	12 (37.50	0.717
Antibiotic therapy (<3 months)	14 (34.1)	5 (55.6)	9 (28.1)	0.231
Digestive therapy (<3 months)	5 (12.2)	2 (22.2)	3 (9.4)	0.299
Interval from hospitalization to ICU admission (days)	2 (0–15)	15 (4–22.5)	1 (0–8)	0.008
ICU admission				0.022
*Via* emergency department	14 (34.1)	0 (0.0)	14 (43.8)	
*Via* ward	21 (51.2)	8 (88.9)	13 (40.6)	
From other hospital	6 (14.6)	1 (11.1)	5 (15.6)	
Cause of ICU admission				0.712
Septic shock	19 (46.3)	5 (55.6)	14 (43.8)	
Respiratory failure	22 (53.7)	4 (44.4)	18 (56.3)	
APACHE II at ICU admission	26 (19.5–34.5)	31 (22–39)	25 (19–32.5)	0.196
SOFA score at ICU admission	12 (7–15)	13 (7.5–15)	12 (7–15.5)	0.752
Mechanical ventilation	37 (90.2)	7 (77.8)	30 (93.8)	0.204
Renal replacement therapy	22 (53.7)	5 (55.6)	17 (53.1)	1.000
Length of hospital stay (days)	53 (24.5–107)	60 (33.5–91.5)	50 (22–129)	0.422
Length of ICU stay (days)	15 (8.5–44.5)	17 (8.5–38)	14.5 (8–45)	0.862
Duration of mechanical ventilation (days)	12 (7–38.5)	12 (9–27)	11.5 (7–42)	0.938
ICU mortality	9 (22.0)	2 (22.2)	7 (21.9)	1.000
In-hospital mortality	15 (36.6)	4 (44.4)	11 (34.4)	0.701

Values are presented as mean (IQR) or number (percentage). CRE, carbapenem-resistant enterobacteriaceae; ICU, intensive care unit, APACHE, Acute Physiology and Chronic Health Evaluation; SOFA, Sequential Organ Dysfunction Assessment.

The details of medications and infection status at fecal sampling are presented in Table 2. The interval from hospitalization to fecal sampling date was longer in CRE carriers [26 days (IQR 22–43) vs. 8.5 days (IQR 3.5–20), *p* = 0.006]. At the time of fecal sampling, 68.3% (*n* = 28) were receiving enteral feeding, and H_2_ blockers or proton-pump inhibitors were administered in 92.7% (*n* = 38) of the cohort. Beta-lactam antibiotics were given in 97.6% of the participants. Carbapenem antimicrobials were administered in 53.7% of the participants, and there was no significant difference between CRE positive and negative groups (66.7 vs. 50.0%, *p* = 0.466). Bacteremia and CDI were noted in 43.9% (*n* = 18) and 12.2% (*n* = 5) of the cohort, respectively. The MDROs constituted 7.3% (*n* = 3) of MRSA and 12.2% (*n* = 5) of VRE infections.

**TABLE 2:** Medications and CRE carrier status at fecal sampling of the participants.

Variables	Total (*n* = 41)	CRE carriers (*n* = 9)	CRE non-carriers (*n* = 32)	*p*-value
Interval hospitalization to sample (days)	15 (5.5–25.5)	26 (22–43)	8.5 (3.5–20)	0.006
Diet				0.228
Nil *per os*	13 (31.7)	1 (11.1)	12 (37.5)	
Enteral nutrition	28 (68.3)	8 (88.9)	20 (62.5)	
**Medications**				
Opioid	25 (61.0)	5 (55.6)	20 (62.5)	0.717
Sedatives	18 (43.9)	3 (33.3)	15 (46.9)	0.706
Norepinephrine	18 (43.9)	6 (66.7)	12 (37.5)	0.147
Vasopressin	6 (14.6)	1 (11.1)	5 (15.6)	1.000
Probiotics	5 (12.2)	0 (0.0)	5 (15.6)	0.568
H_2_ blocker or PPI	38 (92.7)	9 (100.0)	29 (90.6)	1.000
Carbapenem	22 (53.7)	6 (66.7)	16 (50.0)	0.466
Quinolone	18 (43.9)	3 (33.3)	15 (46.9)	0.706
Beta-lactam	40 (97.6)	9 (100.0)	31 (96.9)	1.000
Piperacillin/tazobactam	17 (41.5)	2 (22.2)	15 (46.9)	0.262
Antifungal agent	11 (26.8)	3 (33.3)	8 (25.0)	0.680
Bacteremia	18 (43.9)	5 (55.6)	13 (40.6)	0.471
*Clostridium difficile* infection	5 (12.2)	2 (22.2)	3 (9.4)	0.299
IPA	5 (12.2)	1 (11.1)	4 (12.5)	1.000
**MDROs**				
ESBL-E	11 (26.8)	2 (22.2)	9 (28.1)	1.000
MRAB	4 (4.0)	0 (0.0)	4 (3.1)	0.559
MRSA	3 (7.3)	0 (0.0)	3 (9.4)	0.340
VRE	5 (12.2)	2 (22.2)	3 (9.4)	0.299

Values are presented as mean (IQR) or number (percentage). CRE, carbapenem-resistant Enterobacteriaceae; ICU, intensive care unit; PPI, proton-pump inhibitor; IPA, invasive pulmonary aspergillosis; MDRO, multidrug-resistant organism; ESBL-E, extended-spectrum β-lactamases producing Enterobacteriaceae; MRAB, multidrug-resistant Acinetobacter baumannii; MRSA, methicillin-resistant Staphylococcus aureus; VRE, vancomycin-resistant Enterococcus.

### 3.2. CRE carriers showed gut microbiota composition and metabolic pathway alterations

**FIGURE 1:**
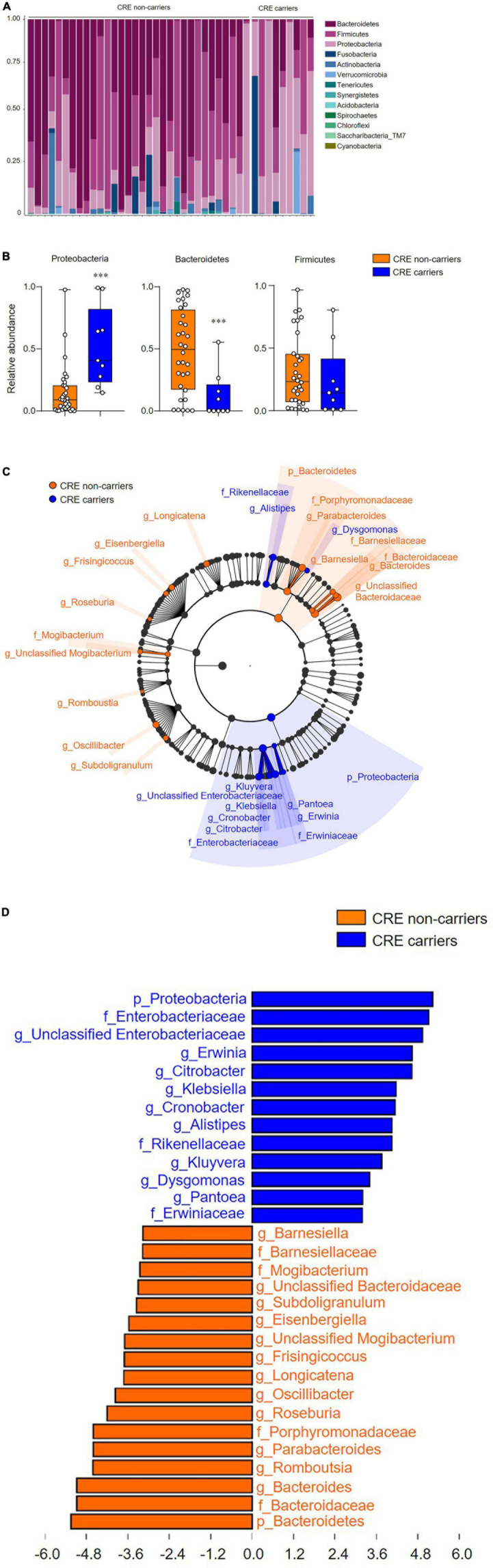
Carbapenem-resistant Enterobacteriaceae (CRE) influences gut microbiota composition and community structure. **(A)** Microbiota composition of feces from CRE non-carriers or carriers at the phylum level. **(B)** Relative abundance of the phyla, Proteobacteria, Bacteroidetes, and Firmicutes. **(C)** Taxonomic cladogram from linear discriminant effect size (LEfSe) analysis. Dot size is proportional to taxon abundance. **(D)** Linear discriminant analysis (LDA) scores obtained from LEfSe analysis of fecal microbiome. An LDA effect size of more than 2 was used as a threshold for the LEfSe analysis. Statistical analyses were performed using the Mann–Whitney *U* test. ^***^p < 0.001.

We first addressed the changes of gut microbiota composition from CRE carriers (Figure 1A). Of note, the relative abundance of the phyla in CRE carriers comprised 52% Proteobacteria, 23% Firmicutes, and 12% Bacteroidetes. In CRE non-carriers, the relative abundance of the phyla comprised 48% Bacteroidetes, 31% Firmicutes, and 15% Proteobacteria. Compared to CRE non-carriers, the gut microbiota from CRE carriers showed an increased proportion of Proteobacteria and decreased proportion of Bacteroidetes (*p* < 0.001; Figure 1B). Additionally, LDA with LEfSe revealed that several bacterial genera were noticeably altered in the gut microbiota of CRE carriers compared with those in non-carriers (Figure 1C). In CRE carriers, the genera *Erwinia*, *Citrobacter*, *Klebsiella, Cronobacter*, *Kluyvera*, *Dysgomonas*, *Pantoea*, and *Alistipes* showed upper 2 LDA scores (Figure 1D) whereas the genera *Barnesiella, Subdoligranulum, Eisenbergiella, Frisingicoccus, Longicatena, Roseburia, Oscillibacter, Parabacteroides, Romboutsia*, and *Bacteroides* showed lower 2 LDA scores.

**FIGURE 2:**
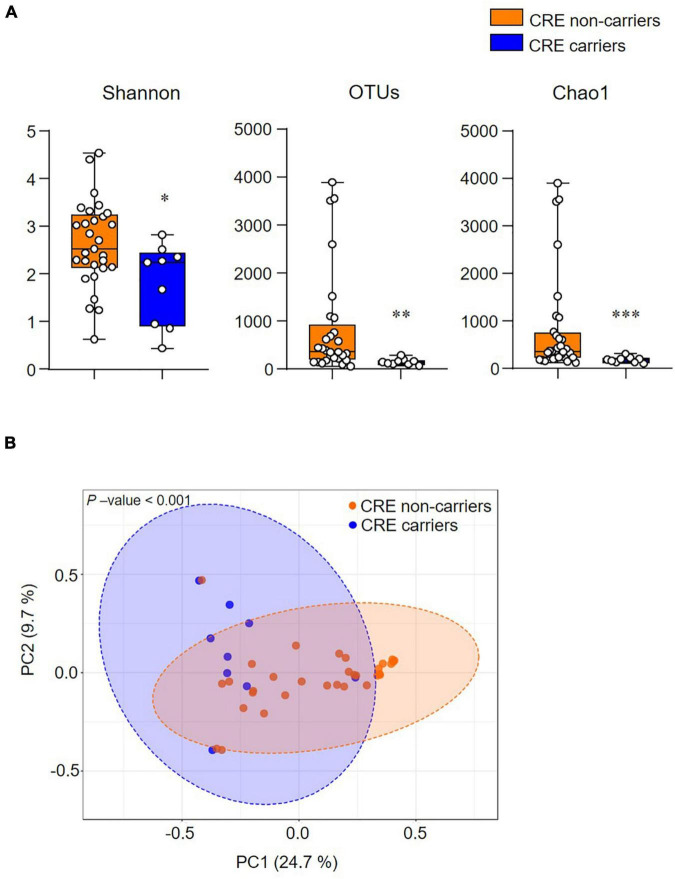
The community structure of the gut microbiome is associated with CRE colonization. **(A)** Alpha-diversity indices (Shannon, Observed OTUs, and Chao1). **(B)** Beta-diversity analysis based on the Bray–Curtis distance. Statistical analyses were performed using the Mann–Whitney *U* test. *p < 0.05, ^**^p < 0.01, ^***^p < 0.001.

The alpha diversity index (Shannon, OTUs, and Chao1) significantly decreased in CRE carriers compared to the CRE non-carriers (Figure 2A). Furthermore, the beta diversity analysis (Bray–Curtis distances) demonstrated that the two groups were clustered significantly apart (*p* < 0.001; Figure 2B).

**FIGURE 3:**
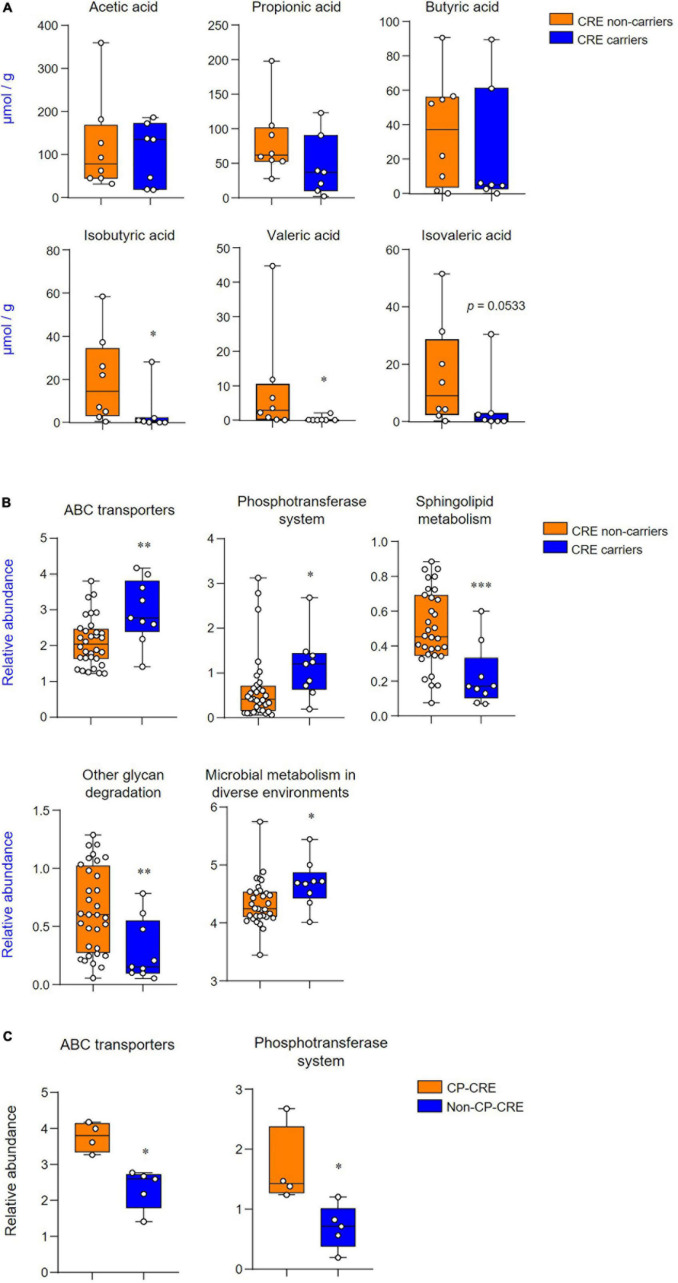
CRE changes associated with the levels of short-chain fatty acids and functional metabolic profile. **(A)** Quantification of acetic, propionic, butyric, isobutyric, valeric, and isovaleric acids derived from the fecal extract. **(B)** Predicted functional profiling of microbial communities based on 16S rRNA sequences. The relative abundances of level 3 KEGG pathway (LDA scores more than 2 are shown); ABC transporters (KO 02010), phosphotransferase system (KO 02060), sphingolipid metabolism (KO 00600), other glycan degradation (KO 00511), and microbial metabolism in diverse environments (KO 01120). **(C)** Predicted functional profiling of microbial communities between CP-CRE and non-CP-CRE. Statistical analyses were performed using the Mann–Whitney *U* test. *p < 0.05, ^**^p < 0.01, ^***^p < 0.001.

The short-chain fatty acid levels of fecal extracts were examined to identify which metabolites were associated with CRE carriers and non-carriers. Among the short-chain fatty acids, the levels of isobutyric acid and valeric acid were significantly decreased in CRE carriers (*p* < 0.05; Figure 3A). PICRUSt-predicted metabolic pathways showed five significant differences in the features between CRE carriers and non-carriers and these included: the KEGG pathway for ATP-binding cassette (ABC) transporters, phosphotransferase systems, sphingolipid metabolism, other glycan degradation, and microbial metabolism in diverse environments (Figure 3B). Among CRE carriers, patients with CP-CRE had significantly increased KEGG pathways for ABC transporters and phosphotransferase systems than those with non-CP-CRE (*p* < 0.05; Figure 3C).

## 4. Discussion

In this study, we compared the gut microbiome in critically ill patients according to the CRE carrier status and found that CRE carriers showed increased and decreased proportions of the Proteobacteria and Bacteroidetes phyla, respectively. The alpha-diversity indices were significantly decreased in CRE carriers as compared with those in CRE non-carriers. Of note, the two groups were clustered significantly in the beta-diversity analysis. Furthermore, we observed that levels of short-chain fatty acids, such as isobutyric acid and valeric acid, had significantly decreased in CRE carriers. In terms of metabolic pathways, CRE carriers showed distinctive features as compared with CRE non-carriers and these included increased ABC transporters and decreased sphingolipid metabolism.

Recent studies demonstrated that CRE carriers had significantly higher number of Proteobacteria and lower prevalence of Firmicutes and Bacteroidetes than CRE non-carriers and healthy controls (Korach-Rechtman et al., 2020; Sindi et al., 2022). The results of our study revealed findings that are aligned with those previous reports. Proteobacteria dominance is associated with increased inflammation (Frank et al., 2007; Shin et al., 2015), which can be attributed to CRE (Lee and Kim, 2011). Another notable finding is the change of the alpha- and beta-diversity indices in the two groups. The findings of this study are consistent with the results of a previous study (Korach-Rechtman et al., 2020), wherein the alpha-diversity indices of gut microbiota were significantly decreased in patients who were CRE carriers as compared to that in non-carriers. Lee et al. (2021) reported alterations of gut microbiota in CRE carriers during fecal microbiota transplantation (FMT): the Shannon diversity index of gut microbiota in recipients significantly increased (*p* < 0.05), and 90% of CRE carriers were decolonized after FMT. Therefore, the restoration of microbiota balance may reduce CRE colonization (Dong et al., 2020).

In critically ill patients, alterations of the gut microbiota can affect patient outcomes. Garcia et al. (2022) demonstrated that the abundance of the Enterococcaceae family was associated with an increased risk of infection and death in the ICU. We further evaluated the microbiome taxonomy abundance at the genus level. We observed that the genera *Erwinia*, *Citrobacter*, *Klebsiella, Cronobacter*, and *Pantoea* were significantly abundant in CRE carriers. These results resemble those in the report of Korach-Rechtman et al. (2020) and revealed an increased abundance of *Enterobacter*, *Erwinia*, *Pantoea*, and *Klebsiella*. Notably, although phylum Bacteroidetes is decreased, genus *Alistipes* is significantly increased in CRE carriers. Some strains of *Alistipes* have antimicrobial resistance to beta-lactam antibiotics (Parker et al., 2020), and these may be associated with the abundance in CRE positive patients. The abovementioned authors suggested that these potentially virulent resident species become a predisposing factor, and consequently lead to host infection. A previous animal study showed that intestinal Enterobacteriaceae colonization was promoted by antibiotic treatment (Ubeda et al., 2010). Therefore, antibiotic therapy may accelerate these alterations of microbiota composition or induce changes in colonization resistance due to the decreased diversity. Furthermore, we observed that the *Barnesiella* and *Bacteroides* genera decreased in critically ill patients with CRE carriers. A previous study demonstrated that oxygen-tolerant bacteria, such as VRE, were suppressed by commensal anaerobic bacteria (Ubeda et al., 2013). In particular, the genus *Barnesiella*, which belongs to the phylum Bacteroidetes, was associated with protection against VRE domination. Similar to the findings of Ubeda et al., the *Barnesiella* species was less abundant in CRE carriers than in non-carriers. Therefore, this species is expected to be involved in restriction of proliferation of CRE as well as VRE.

Short-chain fatty acids are involved in the colonization resistance of antibiotic-resistant pathogens such as VRE or CRE (Keith and Pamer, 2019). According to Sorbara et al. (2019) triggering short-chain fatty acid–mediated intracellular acidification is associated with in inhibiting the overgrowth of antibiotic-resistant Enterobacteriaceae. Furthermore, level of short-chain fatty acid was negatively correlated with *E. coli* within the microbiota in a patient with allogeneic hematopoietic stem cell transplantation. In line with this, previous studies showed that critically ill patients had a decrease in fecal short-chain fatty acids (Yamada et al., 2015; Valdés-Duque et al., 2020). Of note, our results revealed that the major three short-chain fatty acids did not differ significantly between the two groups. It is interesting to note, however, that valeric acid and branched short-chain fatty acid levels, including those of isobutyric acid and isovaleric acid, are reduced in CRE carriers compared with CRE non-carriers. The decreased levels of short-chain fatty acids are associated with infection as follows: isobutyric acid in patients diagnosed with infection upon ICU admission (Valdés-Duque et al., 2020) and valeric acid in patients with recurrent CDI (McDonald et al., 2018). These short-chain fatty acids are mainly produced by the phyla Firmicutes and Bacteroidetes (Rios-Covian et al., 2015). Therefore, low levels of short-chain fatty acids in CRE carriers might be attributable to the changes in the phyla. Furthermore, valeric acid, isobutyric acid, and isovaleric acid are produced by the amino acids proline and hydroxyproline and valine and leucine, respectively (Zarling and Ruchim, 1987; Rasmussen et al., 1988). Therefore, amino acid supplementation could affect the microbiome dysbiosis (Yang et al., 2016).

The KEGG pathway analysis revealed that CRE carriers have a distinctive metabolic pathway, with an abundance of ABC transporters, which are one of the superfamily of integral membrane proteins and are involved in the translocation of various ATP-using substrates (Davidson et al., 2008). Drug resistance against anticancer or antimicrobial agents can be caused by activation of transmembrane proteins that efflux substances from the cells (Choi, 2005). Although enzymatic production is the main mechanism of CP-CRE, efflux pumps constitute an important mechanism of non-CP-CRE (Suay-García and Pérez-Gracia, 2019). Furthermore, we identified that ABC transporters are more abundant in patients with CP-CRE than those with non-CP-CRE. Therefore, these results suggest that ABC transporters play a role in the efflux of carbapenem in the CRE. Another notable finding in the KEGG pathway analysis is that sphingolipid metabolism is significantly decreased in CRE carriers compared with non-carriers. Sphingolipids are structural membrane components, and dysregulated sphingolipid homeostasis is associated with various pathophysiologic conditions, such as inflammatory bowel disease, cancer, or neurodegenerative diseases (Quinville et al., 2021). Brown et al. (2019) reported that the levels of sphingolipids produced by *Bacteroides* spp. are decreased in the stool of the subjects with inflammatory bowel disease, and that sphingolipid-deficiency is associated with intestinal inflammation and barrier dysfunction. Therefore, the sphingolipid signaling pathways may play an essential role in the pathogenesis of inflammatory bowel disease. Furthermore, An et al. (2011) suggested that a sphingolipid-mediated mechanism is utilized by the *Bacteroides* spp. to survive in the stressful intestinal environment. Therefore, the decreased proportion of *Bacteroides* in CRE carriers could potentially result in decreased sphingolipid metabolism.

We extensively evaluated the gut microbiome and the related metabolic pathways of critically ill patients with CRE. Nonetheless, there are several limitations of this study that warrant mention. First, due to the retrospective study design, detailed data of the diet were limited and were not controlled for between two groups. The diet is closely associated with the gut microbiota (Turnbaugh et al., 2009; Maslowski and Mackay, 2011), however, the majority of the patients received commercial enteral nutrition and parenteral nutrition. Second, since no healthy controls were enrolled, we analyzed the microbiome in critically ill patients with or without CRE. The ICU environment and medications might be associated with the similar proportion of Firmicutes between two groups (Korpela et al., 2016; Oami et al., 2019). Third, the interval between hospitalization and fecal sampling date was significantly longer in CRE carriers than in CRE non-carriers. CRE colonization is associated with longer hospitalization, ICU stay, and antibiotic usage (Dong et al., 2020). Most participants were exposed to a broad spectrum of antibiotics, and the number of CRE positive groups was small, so there was no difference in carbapenem usage between the two groups. Fourth, we cannot rule out the possibility that co-infection or co-colonization may have affected the gut microbiota. In the ICU setting, patients with CRE acquisition have multiple co-colonizations with other MDROs (Kang et al., 2019). Therefore, it was difficult to recruit only CRE carriers who did not have other MDROs in our cohort. Fifth, given the relatively small number of study population, it is difficult to generalize the results of our study. However, our study showed the alterations of levels of short-chain fatty acids and metabolic pathways according to CRE carriage. We also showed the significant differences of KEGG pathways related to efflux systems between CP-CRE and non-CP-CRE though we need to validate on large samples. Our study may suggest the role of metabolites in the management of CRE colonization.

## 5. Conclusion

In critically ill patients, CRE carriers demonstrated distinctive features of the gut microbiota and diversity indices. Furthermore, the levels of branched short-chain fatty acids were decreased, and several changes in metabolic pathways, such as increased levels of ABC transporters and decreased sphingolipid metabolism, were observed in CRE carriers. Further research is needed to ascertain whether amino acid supplementation or FMT could suppress the pressure of CRE acquisition.

## Data availability statement

The datasets presented in this study can be found in online repositories. The names of the repository/repositories and accession number(s) can be found below: https://www.ncbi.nlm.nih.gov/, PRJNA918541.

## Ethics statement

The studies involving human participants were reviewed and approved by the Institutional Review Board of Asan Medical Center. The patients/participants provided their written informed consent to participate in this study.

## Author contributions

MB and JH conceived and designed the study. MB, W-YK, and JH collected the primary data. SK and M-NK conducted the data analyses and interpreted the results. MB, SK, and JH drafted the initial manuscript. All authors revised the manuscript for important intellectual content and approved the final manuscript for publication.
